# Editorial: Celebrating Twenty Years of the Brazilian Symposium on Cardiovascular Physiology

**DOI:** 10.3389/fphys.2017.00166

**Published:** 2017-03-21

**Authors:** Camille M. Balarini, Valdir A. Braga

**Affiliations:** ^1^Department of Physiology and Pathology, Health Sciences Center, Federal University of ParaibaJoao Pessoa, Brazil; ^2^Biotechnology Center, Federal University of ParaibaJoao Pessoa, Brazil

**Keywords:** cardiovascular physiology, cardiovascular diseases, hypertension, vascular function

This research topic is dedicated to the celebration of 20 years of the Brazilian Symposium on Cardiovascular Physiology. In 1996 groups from the School of Medicine of Ribeirao Preto, University of Sao Paulo (FMRP-USP) and from the Federal University of Sao Paulo (UNIFESP) joined together to discuss cardiovascular physiology. In subsequent editions of the meeting, the participation of other groups from all over the country has grown and acquired the status of a national symposium. The participants now agree that the symposium should be itinerant and that the chair group is responsible for its organization. In 2016, we proudly reached the 20th edition of the Brazilian Symposium on Cardiovascular Physiology. It is certainly a memorable date and a great opportunity to share the accomplishments of Brazilian groups in the field of cardiovascular physiology.

The groups devoted to investigate cardiovascular physiology in Brazil descended, in a direct or indirect manner, from the Argentinian physiologist and Nobel Prize winner Bernardo Houssay. One of his disciples, Miguel Covian, was invited to be the chair of the Department of Physiology in the School of Medicine of Ribeirao Preto when it was funded in the 1950's. At the same time, the physicians at Federal University of Rio Grande do Sul started a collaborative work with Bernardo Houssay's and Braun Menendez's groups. That historical perspective is presented in this issue in the article by Vasquez. In his paper, he also shares the contributions of Professor Eduardo M. Krieger to the development of cardiovascular physiology in Brazil.

The etiology of cardiovascular and metabolic diseases reveals the involvement of different genetic, environmental, nutritional and behavioral aspects. In this issue, Costa-Silva et al. discuss the role of maternal diet on the development of cardiometabolic diseases. The authors point out that epigenetic alterations can be, at least in part, responsible for the increased risk of developing cardiometabolic problems. The authors discuss in detail the manner in which maternal protein undernutrition or overnutrition during the perinatal period can increase the risk of cardiovascular and metabolic diseases.

It is important to highlight that the success of the experimental design for investigating cardiovascular physiology depends on the availability of suitable experimental models. In this context, Crestani reviews the effects of acute and chronic emotional stress in cardiovascular function. The author focused on the cardiovascular responses observed in different animal models of emotional stress. Considering the impact of stress in the cardiovascular system in humans, this is certainly a promising area of research.

The influence of the autonomic nervous system in cardiovascular function is remarkable. Thus, different research groups in Brazil are devoted to investigating such influence in health and disease. In the present issue, Accorsi-Mendonça et al. present a historical retrospective on the characteristics of rostral ventrolateral medulla (RVLM) presympathetic neurons and discuss the concept that those cells work as pacemakers for the generation of the sympathetic activity. Interestingly, physical activity can modulate central areas involved in autonomic control of the cardiovascular system. In this context, Raquel et al. show that swimming modulates nitric oxide (NO) availability and glutamatergic neurotransmission in the RVLM, contributing to a decrease in sympathetic activity and an increase in baroreflex control of blood pressure (BP). This provides insight and support to the idea that physical activity should be included when treating hypertensive patients.

Pharmacological modulation of the autonomic nervous system is an important target to treat cardiovascular diseases (CVD). Alternative experimental approaches in this field include the search for new natural and synthetic compounds. In this regard, Pinto et al. observed that bombesin, a peptide isolated from frog skin, increases blood pressure and renal sympathetic nerve activity (RSNA) when administered into the RVLM of normotensive and spontaneously hypertensive rats (SHR). In addition, Silva et al. show that ivabradine reduced resting heart rate and blood pressure, with no effects on cardiovascular reflexes or RSNA.

Other important group of neurons involved in autonomic cardiovascular control are present in the paraventricular nucleus of the hypothalamus (PVN). This region possesses reciprocal communications with the nucleus tractus solitarii (NTS) and synapses with both the RVLM itself and the subfornical organ (SFO), which receives information from directly from the blood stream since it lacks a blood brain barrier. In this context, De Melo et al. hypothesize that the decrease in ovarian hormones during menopause blunts oxytocin expression and signaling in pre-autonomic PVN neurons, leading to baroreflex impairment, autonomic imbalance and arterial hypertension. This reinforces clinical findings that women are more prone to develop CVD after menopause than men of the same age and provides experimental data to further support hormonal reposition therapies.

Considering that an increase in blood pressure variability (BPV) is an indicative of poor prognosis in cardiovascular outcomes, Freitas et al. demonstrate that increased BPV prior to the onset of chronic kidney disease can reduce renal blood flow, increase renal vascular resistance and increase uraemia and glomerulosclerosis, exacerbating renal dysfunction. In this scenario, the authors suggest that increased BPV may be considered as a marker for target-organ damage.

Recently, the gut microbiota has gained attention as it can be involved in the onset of diverse pathological states, including the development of CVD. In this context, de Brito-Alves et al. show evidence, from both experimental and clinical approaches, that the use of polyphenols and probiotics reduces blood pressure and improves cardiovascular function. In agreement with this, Klippel et al. present an experimental study in which the administration of kefir (a probiotic composed by different bacteria such as *Lactobacillus kefiranofaciens, Lactobacillus kefir*, and *Candida kefir*) to SHR resulted in the amelioration of vagal and sympathetic imbalance, improvement of baroreflex sensitivity and a reduction in blood pressure. Taken together, these reports emphasize the potential of probiotics as adjuvants on CVD treatment.

Despite the fundamental role played by the central nervous system (CNS) in cardiovascular control, the peripheral control of blood flow to target organs in response to specific organ demands presents an important role in BP homeostasis. This is particularly evident during sepsis and septic shock, when a massive vasodilation causes a remarkable reduction in blood pressure, with a high mortality rate. During this state, there is also a vascular hyporesponsivity to vasoconstrictors, limiting therapeutic options. Thus, Pernomian et al. used an experimental model of sepsis to evaluate the participation of C-type natriuretic peptide on this response. The authors suggest that this peptide is involved in hyporeactivity to vasoconstrictors in aorta, revealing a novel potential target for septic shock.

In the present issue, Costa et al. provide a relevant review on the vascular effects of neuronal nitric oxide synthase (nNOS). It is important to highlight that, although endothelial NOS (eNOS) is considered the main NOS isoform in vessels, nNOS is an important source of both NO and hydrogen peroxide (H_2_O_2_), two endothelium-dependent vasodilators. Imbalances in nNOS expression and/or activity have been described in arterial hypertension and atherosclerosis, corroborating the idea that this isoform is particularly relevant for proper vessel function. Nitric oxide signaling can be modulated by physical activity not only in the CNS as previously discussed but also in the periphery. In this context, Macedo et al. evaluated the participation of NO in vascular tone adjustments in response to low-intensity resistance training. They observed increased expression of eNOS and nNOS, culminating in an improvement of vascular function, increase in baroreflex sensitivity and reduction in blood pressure.

Additionally, a further guardian of vascular homeostasis is perivascular adipose tissue (PVAT), which can secrete vasoactive substances. In this context, Victorio et al. comparatively evaluated the effects of PVAT from abdominal and thoracic aorta. The authors observed functional regional differences along the aorta, with a greater production of vasodilators in thoracic vs. abdominal PVAT.

Despite the efforts of diverse research groups in Brazil and abroad, CVD still is the leading cause of death worldwide. The efficient function of the cardiovascular system depends to a large degree on good cardiac function, which may be impaired after myocardial infarction (MI), for example. In this issue, Santana et al. compare two different techniques to induce MI in rats. Patterns of gene expression were seen to differ between the two methods. This study certainly contributes to the standardization of suitable experimental models that will allow new approaches to prevent and treat MI. For instance, Manchini et al. used low-level laser therapy to improve left ventricular systolic function. Although the results are promising, the beneficial effects seem to be transient and long-term studies are needed. Heart failure is commonly observed after MI and this compromises not only the function of the heart *per se* but also leads to damage in target organs. It was observed by Arruda-Junior et al. that rats with heart failure presented impaired renal function (fluid retention, reduction in glomerular filtration rate, increase in protein excretion), which was preserved by the inhibition of dipeptidyl peptidase IV. de Melo et al. provide us with another example of beneficial outcomes from physical exercise. The authors evaluated the effects of exercise training in a model of right ventricular remodeling and found that it attenuated myocardial remodeling and improved right ventricular function.

Different strategies to deal with CVD often come from the discovery of new physiological pathways. In this context, Xu et al. reviewed the role played by ADAM17 (A disintegrin A metalloprotease 17) in the cardiovascular system as well as in CNS areas involved in cardiovascular control. Although it is a very promising target molecule, the diversity of its substrates (this enzyme is involved with more than 70 different substrates) including inflammatory substances, have slowed the progression of translational studies.

As highlighted here, based on the quality of the different studies presented, the initiative of creating the Brazilian Symposium on Cardiovascular Physiology 20 years ago has contributed enormously to the development of research on cardiovascular physiology in Brazil. The country now holds more than 20 different research groups established from the north to the south of the country producing worldwide recognized science. Most laboratories are well-equipped with cutting-edge technology allowing in-deep investigations into cardiovascular phenomena. The main limitations currently faced by Brazilian researchers are the strict rules governing the importation of research goods (such as chemicals, reagents, live animals, viruses for gene transfer and general lab equipment) and the uneven distribution of research funding across the country. In conclusion, we would like to thank very much all the Brazilian research groups who attended to the 20th Brazilian Symposium on Cardiovascular Physiology and the reviewers who generously agreed to review the manuscripts presented in this issue.

## Author contributions

CB and VB participated in all stages during the elaboration of this manuscript. Authors read and approved the final version.

### Conflict of interest statement

The authors declare that the research was conducted in the absence of any commercial or financial relationships that could be construed as a potential conflict of interest.

